# Protocols for 16S rDNA Array Analyses of Microbial Communities by Sequence-Specific Labeling of DNA Probes

**DOI:** 10.1100/tsw.2003.44

**Published:** 2003-07-01

**Authors:** Knut Rudi, Janneke Treimo, Hilde Nissen, Gerd Vegarud

**Affiliations:** MATFORSK Norwegian Food Research Institute, As, Norway

**Keywords:** rRNA, rDNA, DNA array, microbial community

## Abstract

Analyses of complex microbial communities are becoming increasingly important. Bottlenecks in these analyses, however, are the tools to actually describe the biodiversity. Novel protocols for DNA array-based analyses of microbial communities are presented. In these protocols, the specificity obtained by sequence-specific labeling of DNA probes is combined with the possibility of detecting several different probes simultaneously by DNA array hybridization. The gene encoding 16S ribosomal RNA was chosen as the target in these analyses. This gene contains both universally conserved regions and regions with relatively high variability. The universally conserved regions are used for PCR amplification primers, while the variable regions are used for the specific probes. Protocols are presented for DNA purification, probe construction, probe labeling, and DNA array hybridizations.

There has been a tremendous development of DNA array-based applications[1] since the pioneer work by P. Brown[2]. Generally, DNA arrays are used either for global gene expression analyses or for screening of genes present or absent in a given genome. These arrays may include several thousand different probes in a single analysis.

In this paper we focus on the use of DNA arrays for analyses of microbial communities. These types of analyses require the processing of many samples in parallel with relatively few probes[3]. A major issue in these analyses is the signal-to-noise ratios, since single nucleotide polymorphisms (SNPs) are commonly targeted[4]. We have developed a novel approach that combines the specificity obtained by sequence-specific labeling of DNA probes with the possibility of DNA array hybridization (Fig. 1). This approach gives a very high signal-to-noise ratio[5,6].

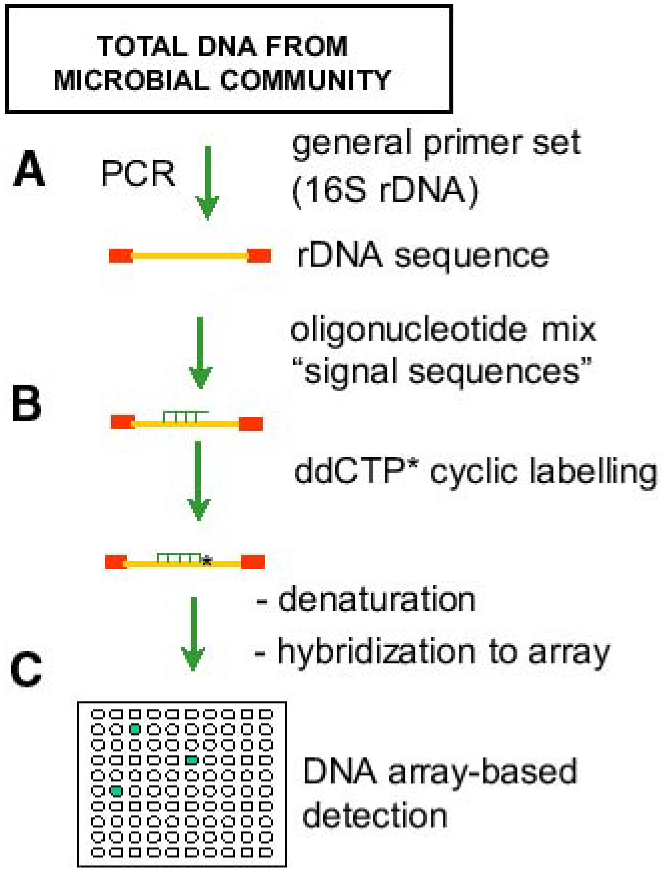

**FIGURE 1.** Schematic representation of the DNA array method. Total DNA from a microbial community is PCR amplified with universal 16S rDNA primers (A). Then, specific probes are added and labeled sequence specifically (B). Finally, the probes are hybridized to their respective complements on a solid phase (C).

The genes encoding ribosomal RNA are present in all living organisms. These genes include both universally conserved regions and regions with relatively high variability[7] (Fig. 2). The conserved regions are used for the localization of PCR amplification primers, while the variable regions are used for the specific probes.

Protocols are given for the purification of DNA from microbial communities, sequence-specific labeling of the DNA probes, and DNA array hybridization. Examples are shown both for membrane- and glass slide–based arrays.

## PROBE CONSTRUCTION

The probes for sequence-specific labeling are constructed from the criteria that the melting temperature of the probes should be between 60 and 70°C, and the size between 20 and 30 nucleotides. The probes are constructed so that the nucleotide following at the 3′-end is a discriminatory cytosine.

Alignments are generated by using sequence alignment software, e.g., Clustal X[8]. The alignments are then manually edited using the programs such as BioEdit[9]. The discriminatory substitutions are subsequently identified manually in the alignments.

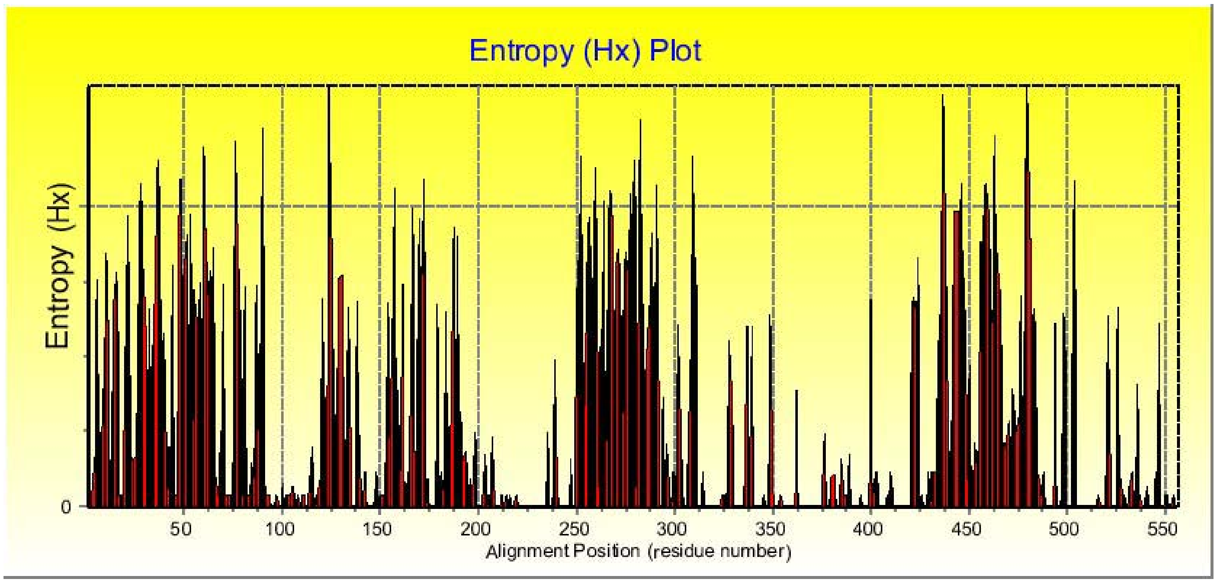

**FIGURE 2.** Entropy plot for a part of the 16S rDNA gene. The plot is based on 150 partial 16S rDNA sequences obtained from microorganisms on fish samples. The regions with high entropy are variable, while the regions with low entropy are conserved. (Nissen et al., manuscript in preparation.)

## DNA PURIFICATION AND PCR AMPLIFICATION

Samples containing approximately $10^8$ bacterial cells are disrupted mechanically with glass beads (106 µm, Sigma, Steinheim, Germany) in a Fast-Prep bead beater (Bio 101, La Jolla, California). The treatment is done for 2 × 20 s at maximum speed. The reason for using mechanical lysis is that this treatment gives the most uniform result (equal lysis for all bacteria in the sample). Subsequently, the DNA is purified with the DNeasy tissue kit (Qiagen, Hilden, Germany) following the manufacturer's recommendations, eluting the DNA in a 100-µl volume.

The genes encoding the small subunit ribosomal RNA (16S rDNA) are then amplified through the application of primers targeted to universally conserved regions of the gene. Primers located at position 10–34 and 1485–1507 relative to the published 16S rRNA sequence for *E.* coli[10] worked well in our applications[5]. The primers have the following sequence; forward 5'-TGG CTC AGA TTG AAC GCT GGC GGC-3' (KR1), reverse 5'-TAC CTT GTT ACG ACT TCA CCC CA-3' (KR2).

We use the GeneAmp 9600 PCR system (Applied Biosystems, Norwalk, Connecticut) for the thermocycling. The 50-µl reaction contains 10 pmol of primers, 200 µ*M* of each deoxynucleotide triphosphate (dNTP), 10 m*M* Tris-HCl (pH 8.8), 1.5 m*M* $MgCl_2$, 50 m*M* KCl, 0.1% Triton X-100, 2 U DynaZyme thermostable DNA polymerase (Finnzymes OY, Espoo, Finland) and 1 to 5 µl of purified DNA. The PCR is initiated with a denaturation step at 94°C for 4 min. This is followed by 30 cycles with the following denaturation, annealing, and synthesis parameters; 95°C for 30 s, 55°C for 30 s, and 72°C for 90 s (for the primer set KR1 and KR2). An extension step for 7 min at 72°C is included at the end of the PCR.

## SEQUENCE-SPECIFIC LABELING

The nucleotides in 20 µl of the reaction are dephosphorylated after the amplification through the addition of 4 U shrimp alkaline phosphatase (U.S. Biochemical Company, Ohio) and incubated at 37°C for 30 min, followed by 10 min at 95°C to inactivate the enzymatic activity. The treated products are then used for the cyclic labeling reaction. The cyclic labeling conditions are the following: 1 × Thermo Sequenase reaction buffer (Amersham Pharmacia, Little Chalfont, Buckinghamshire, England), 10 pmol of each specific probe, 100 pmol ddNTP (except ddCTP) (Roche Molecular Biochemicals, Mannheim, Germany), 100 pmol Tamra-ddCTP (for the DNA microarray hybridization) or fluorescein-12-ddCTP (for the membrane-based hybridizations) (Perkin Elmer, Boston, Massachusetts), 16 U Thermo Sequenase DNA polymerase (Amersham Pharmacia), and 22 µl phosphatase-treated PCR product in a final volume of 60 µl. The labeling is done using the following parameters: 95°C for 15 s, 50°C for 15 s, and 60°C for 1 min for 10 to 25 cycles.

## DNA ARRAY HYBRIDIZATION

Two different platforms are used in the DNA array experiments. The membrane-based platform includes both a chromogenic and a fluorescence detection. The microarray platform is based on glass slides, and enables detection of multiple colors. The data are analyzed with Array-Pro Analyzer 4.0 (Amersham Pharmacia), or corresponding software.

### Membrane-based Analyses

Fifty pmol of primers, complementary to those used in the labeling reaction, are spotted on GeneScreen Plus nylon membranes (Perkin Elmer) and crosslinked for 25 min with a UV transilluminator (Model TL33, UVP Inc., San Gabriel, California). The time needs to be optimized for other crosslinkers. The membranes are prehybridized in 0.5 *M* $Na_2HPO_4$ (pH 7.2) and 1% SDS for 2 h. The labeled probes are added to 300 µl of 1 × SSC and 6% PEG 6000 and then heated to 80°C for 5 min. The hybridization is done overnight at room temperature with agitation in a Cross Blot Dot Blot hybridization chamber (Sebia, Moulinaux, France). Subsequently the membranes are rinsed in 1 × SSC, 1% SDS for 5 min, then for 5 min in 0.1 × SSC, 0.1% SDS, and finally 30 min in 0.1 *M* Tris-HCl pH 7.5 and 0.15 *M* NaCl. The fluorescein is then either detected by direct fluorescence (Fig. 3A) through scanning the membranes, e.g., with a confocal laser scanner (Typhoon Variable Imager scanner, Amersham Pharmacia plc), or the protocol is continued for chromomeric detection. The membranes are then blocked for 2 h in antibody buffer containing 1% skimmed milk (Difco) (blocking buffer). Next, blocking buffer containing 1/500 antifluorescein HRP-conjugate is added and the hybridization continued at room temperature for 2 h. Finally, the membranes are rinsed for 30 min in antibody buffer and the signals are detected with 4-CN Plus chromogenic substrate according to the manufacturer's recommendations (Perkin Elmer) (Fig. 3B).

### DNA Microarray Analyses

SigmaScreen™ (Sigma) or CMT-GAPS™ (Corning Inc., Corning, New York) coated slides for microarrays may be used for the DNA array hybridization experiments. One µl (100 pmol/µl) of the complementary primers containing a 15-mer poly T tail at the 5'-end is spotted on the slide. The slides are then treated according to the respective manufacturer's instructions.

The hybridizations are done overnight under agitation in room temperature using 50 to 100 µl of the ArrayHyb™ LowTemp Hybridization buffer (Sigma) and from 1 to 10 µl of the labeling mixture. One pmol of CY5 5' labeled probes, the same as the probes used in the cyclic labeling, are added to each sample as an internal control for normalization. One slide is divided into independent reaction chambers using a homemade silicone rubber mask. The washing is performed following the recommendations for ArrayHyb™ LowTemp Hybridization buffer (Sigma). Finally, the fluorescence is detected directly (Fig. 3C ).

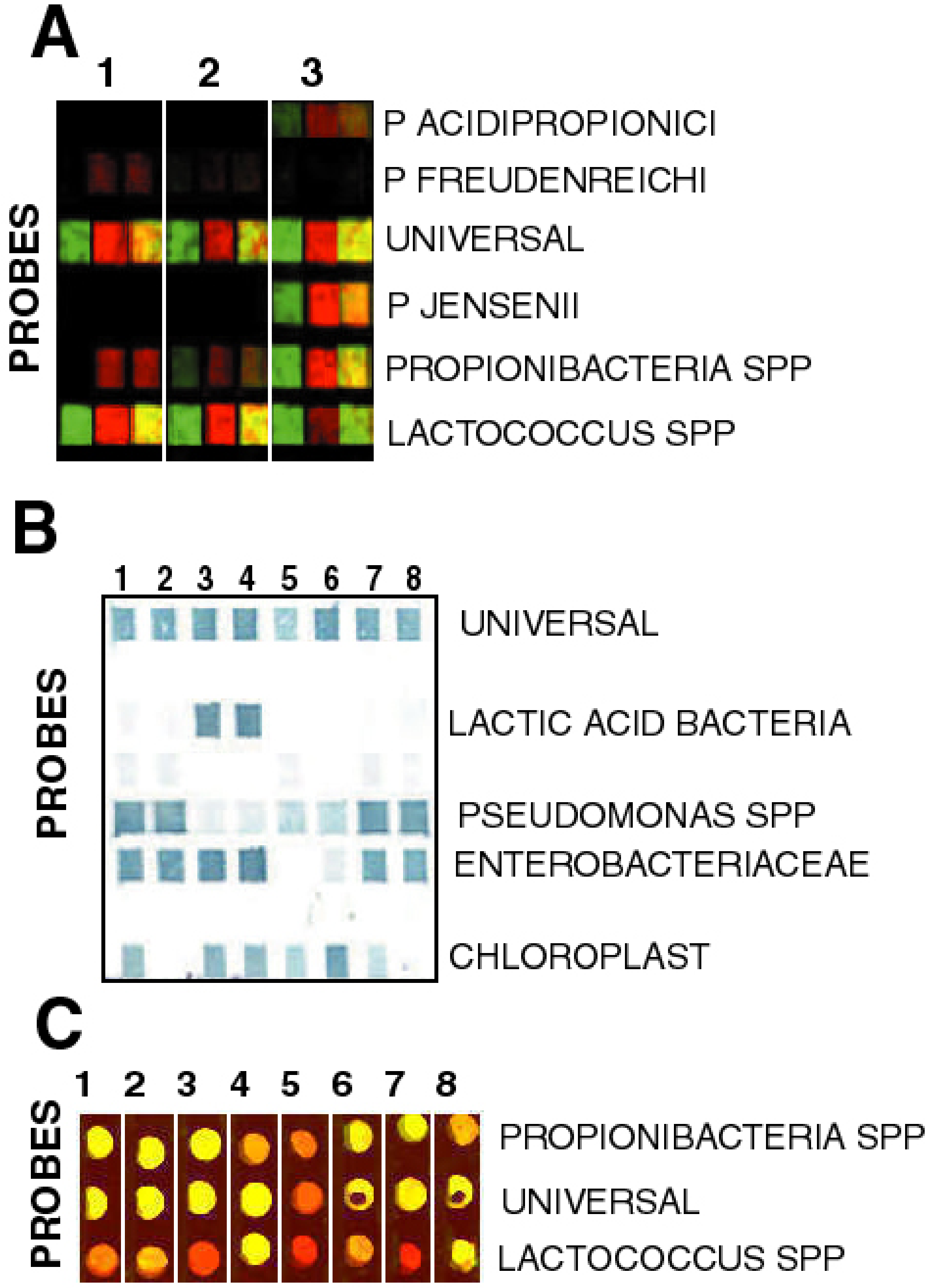

**FIGURE 3.** Examples of detection formats. (A) Direct fluorescence membrane-based detection; the fluorescence from a 24-h sample (green) was compared to that of a sample after 48 h incubation (red). The last column shows an overlay of the two colors. The signals were obtained using a Typhoon model 8600 scanner (Amersham Pharmacia). The following samples were tested: 1 and 2 — different mixtures of *Lactococcus lactis* subsp. L2 and *Propionibacteria freudenreichi* P59; 3 — mixture of *Lactococcus lactis* subsp. L2, *Propionibacteria freudenreichi* P59, *Propionibacteria acidipropionici* ATCC 4965 and *Propionibacteria jensenii* 9614. (B) Chromogenic membrane-based detection; the intensity of the blue color represents the relative amount of a given target. The samples represent the communities identified in eight different salad batches. (C) Glass slide multicolor detection. The direct fluorescence from the sequence-specific labeling (green) is determined relative to an internal control probe (red). The samples 1–8 show different mixtures of *Lactococcus lactis* subsp. L2 and *Propionibacteria freudenreichi* P59.

## APPLICATIONS

Three different systems for the application of the 16S rDNA array analyses are presented. The approach has been used to analyze cyanobacterial communities in water, to analyze microorganisms on vegetables, and finally in DNA array analyses of *Propionibacteria* spp. and *Lactococcus* spp.

### Cyanobacterial Communities in Water

Eight planctonic communities developing in lakes characterized by relatively low algal biomass (mesotrophic), and in lakes with corresponding high biomass (eutrophic) were selected. Ten probes were established to determine the relative abundance of the discernible cyanobacteria encountered in the selected lakes. The probes were generally specific for their targets, as determined through analyses of clone cultures. Reproducible abundance profiles were established for the lakes investigated in the subsequent analyses of natural cyanobacterial communities. The results from the genetic analyses were then compared with information from the standard hydrobiological and hydrochemical analyses. Qualitatively, there were relatively good correlations between the groups of organisms (*Nostoc*, *Microcystis*, and *Planktothrix*) found in the different lakes. The correlations were lower for the quantitative date. This may, however, be due to differences in the sample processing. The conclusions from these comparisons are that the genetic abundance profiles obtained by the 16S rDNA analyses may form a foundation for separating and quantifying genetically distinct groups of cyanobacteria in their natural habitats[3].

### Microbial Communities on Vegetable Salads

The description of microbial communities in ready-to-eat vegetable salads in modified atmosphere was used as the experimental model. Comparisons were made with respect to the effect of storage (up to 12 days) at different temperatures and with respect to the geographic origin of the crisphead lettuce (Spanish or Norwegian). The lettuce was the main salad component. The conclusion drawn from this work was that the DNA array method gave an accurate description of the microbial communities. *Pseudomonas* spp. dominated the microbial flora both in the salad with Norwegian and with Spanish lettuce, before storage and after storage at 4°C. In salad containing Norwegian lettuce the *Pseudomonas* population also dominated after storage at 10°C. In salad containing Spanish lettuce, *Enterobacteriaceae* and lactic acid bacteria dominated the microbial community after storage at 10°C. *Enterobacteriaceae* bacteria were also abundant in the salad with Spanish lettuce after storage at 4°C as well as before storage. The practical implication of these results is that the microbial communities in ready-to-eat vegetable salads can be diverse, and that the microbial composition is both dependent on the origin of the raw material and on the storage conditions[5].

### DNA Array Analyses of *Propionibacteria* and *Lactococcus*

We addressed whether the inclusion of a CY5-labeled internal standard would remove the sample to sample variance due to differences in hybridization conditions for the samples tested. The dilutions of the competitor relative to constant amounts of *Propionibacterium freudenreichii* ISU P59 and *Lactococcus lactis subsp. lactis INF L2* DNA were analyzed in our model system. When analyzing the signal from the sequence-specific labeling (the Tamra-ddCTP signal) independently from the internal control (the CY5 signal), a relatively high variance in the assay was obtained. The variance was probably due to a combined effect of slide to slide variation and differences in the crosslinking of the spotted probes to the slides. However, when analyzing the ratio between the sequence-specific labeled probe and the internal control, most of the variance in the experiment was removed. The resulting assay had relatively low standard deviations[11].

## CONCLUSION

A flexible DNA-based assay for analyses of microbial communities has been developed. The concept was tested on both food and environmental systems. The approach presented can easily be adapted to the analyses of other microbial communities.

## ACKNOWLEDGEMENTS

This work was financed by a research levy on certain agricultural products and grants from the Norwegian Research Council.
